# Calcium Nodules as a Proxy for Quaternary Paleoclimate Change on China’s Loess Plateau

**DOI:** 10.1371/journal.pone.0143928

**Published:** 2015-12-03

**Authors:** Wenming He, Hongming He, Mingyong Zhu

**Affiliations:** 1 School of Chemistry and Environment, Jiaying University, Meizhou, Guangdong, China; 2 State Key Laboratory of Soil Erosion and Dryland Farming on Loess Plateau, Northwest Agriculture & Forestry University, and Institute of Soil and Water Conservation; Chinese Academy of Sciences & Ministry of Water Resources, Yangling, Shaanxi, China; 3 Department of History and Sociology, Minnan Normal University, Zhangzhou, Fujian, China; Universidade de Vigo, SPAIN

## Abstract

Different proxies have been used to investigate Quaternary paleoclimate change. Here, we used weathering of calcium nodules in paleosols on China’s Loess Plateau as a proxy for Quaternary paleoclimate changes to provide an alternative indicator of these changes. Paleosol and carbonate nodules were collected from Luochuan and Lantian counties in Shaanxi Province, China. We found that this approach allowed quantitative reconstruction of temperature, rainfall, soil mineral composition, and the effects of weathering and leaching. The changes in carbonate content in the loess and paleosol sequences were controlled by alternating dry and wet climatic conditions. Nodule formation conditions were directly affected by the leaching and migration of elements. The loess and paleosol sequences developed calcium nodules, and their formation was closely related to the rainfall and leaching characteristics of the paleoclimate. The paleoclimate and soil minerals affected the vegetation types and directly influenced changes in the soil. During formation of the calcium nodules, the surface vegetation evolved slowly, and the number of species and quantity of vegetation both decreased.

## Introduction

Quantitative reconstruction of paleoclimates and paleo-environments, including vegetation communities, is important for studying global climate change, but also provides a clearer understanding of the mechanisms involved in climate change and a valuable historical backdrop for studying future climate change. Soil-formation processes are particularly affected by climatic and hydrologic changes, and these changes are revealed by variations in the characteristics of paleosols [1,2]. In arid and semi-arid regions, older soils typically contain progressively greater amounts of pedogenic carbonate [3]. In paleosol classification, such calcrete soils have been variously considered to be aridisols, calcisols, or paleoaridisols [4–6].

Since the 1970s, researchers have used a variety of physical, chemical and biological indicators to study paleoclimate. For example, topsoil iron samples have been used to quantify the relationship between the total iron content and temperature [7]. In addition, it was possible to determine the soil weathering intensity and eliminate the influence of soil burial and subsequent diagenesis using the chemical index of alteration without potassium (CIA-K) index and regional annual precipitation; for example, the CIA-K index has been applied to investigate rainfall in the Paleozoic to Cenozoic eras [8–10]. The CIA value with potassium (CIA+K) of modern brown soil profiles has been correlated with modern air temperatures and precipitation [11]. It was possible to derive annual-resolution sensitivity in the relationship between weathering during pedogenesis and an index of chemical weathering [12].

Calcium carbonate deposition tends to occur under arid, semi-arid, and semi-humid climate conditions at certain depths in the soil profile. For example, calcium carbonate is a primary constituent of the loess–paleosol sequences on China’s Loess Plateau, and its content and primary dissolution and precipitation, as well as the formation of calcium silicate, are closely related to the chemical weathering intensity, which, in turn, is determined by climatic conditions [7, 11]. Carbonate is mobile in the supergene environment and changes to its migration tend to be restricted by atmospheric precipitation (which controls downward leaching) and temperature (which controls evaporation and upward movement). It is possible, therefore, to infer paleoclimatic conditions by studying secondary carbonates that were derived from weathering and other alterations of primary native carbonates in the soil. Pedogenic carbonate develops during loess and soil development in response to precipitation, *in situ* leaching, and the biological functions of the soil, which include the effects of both plants and soil microbes [13].

The Loess Plateau in China’s Shaanxi Province underwent approximately 22 Ma of continuous loess deposition [14], and therefore contains a large amount of paleo-environmental information in its strata. Long-term regional and global climate change modeling could be improved by a better understanding of the underlying mechanisms, which can be determined by studying the calcium nodules in soil pores of the paleosols, as well as by studying carbon isotopes in the carbonate matrix [15]. From a biological perspective, the Luochuan loess profile records climate change over a long period of time, with excellent preservation. In addition, the deep Quaternary sediment profile is considered to contain one of the best-preserved long-term records of global climate change [16,17]. The loess and paleosol carbonates include native, clastic carbonates, and secondary carbonates [18]. The loess carbonate content and the size of the individual nodules reflects the chemical weathering intensity, the degree of leaching, and changes in the paleo-rainfall and paleoclimate [19].

Using the chemical characteristics of the calcium nodules, researchers can obtain evidence on the climate-controlled genesis of the calcium nodules and changes in the paleo-environment. In the present study, our goal was to quantitatively reconstruct temperature and rainfall regimes during the Quaternary Period in China’s Loess Plateau, particularly with respect to weathering and leaching of the calcium nodules. Our research also revealed the typical evolution of erosion morphology in terms of tectonic, climatic, and anthropogenic changes. Finally, our research provides insights into the dynamics of erosion on the Loess Plateau and a greater understanding of its environmental effects in the middle reaches of the Yellow River, which runs through the plateau.

## Materials and Methods

No specific permissions were required for our experiment, because the study site belongs to the Institute of Soil and Water Conservation, Chinese Academy of Sciences and is only used for scientific research. In addition, our field studies did not involve endangered or protected species.

### 2.1. Data sources

**Fig 1:**
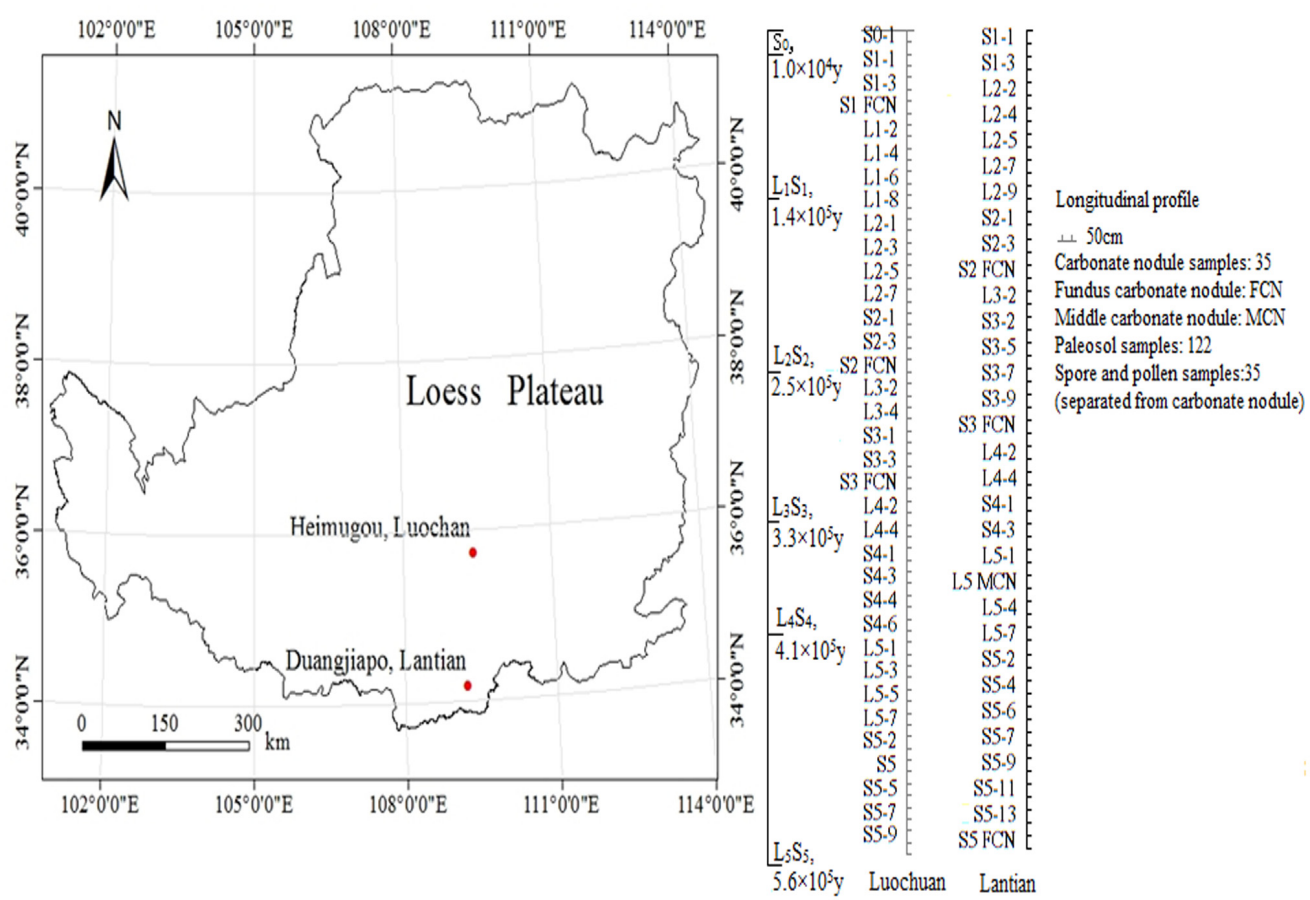
Location of the sample sites where the carbonate nodules and paleosol samples were obtained.

Samples of the paleosol, loess layers and the carbonate nodules were collected in paleosol profiles (S0-S5) and loess profiles (L1-L6) in Luochuan county (42 m in depth, at Heimugou) and Lantian county (37.5 m in depth, at Duanjiapo), both of which are on the Loess Plateau of China (Fig 1). The paleosol and loess were sampled at intervals of 20 to 50 cm, for a total of 84 samples in Luochuan and 75 samples in Lantian. Carbonate nodules were randomly distributed in the paleosols of the Luochuan and Lantian profiles. However, they were more abundant in the Lantian profile, both in terms of the number and thickness of the nodules. We obtained a total of 35 carbonate nodules at each site, and we further categorized them into fundus carbonate nodules and middle carbonate nodules (Table 1) [20].

**Table 1:** Paleosol mineral composition and classification.

*Classification*	*Mineral species*
Light mineral density rho (x < 2.5)	Calcite, quartz, feldspar, mica, quartz > 50%
Medium gravity mineral (2.50 < x < 4.0)	Dolomite, gypsum, chalcedony > 43%
Heavy mineral (x > 4.0)	Many more types (more than 30), 4–7%

### 2.2 Laboratory analysis

To determine the pH and redox potential (Eh) of the soil samples, the samples were air-dried for 48 h, and then milled in a porcelain mortar to pass through a #60 mesh. We dissolved 5 g of each sample in 25 mL of distilled water to remove CO_2_ and then determined the pH and Eh of the supernatant using a pHS-3 acidity meter. The average relative deviations were < 2% for pH and < 3% for Eh (*n* = 8 samples at each site). The total element contents of the soil samples and carbonate nodules were determined after oven-drying the samples for 30 min at 313 K. The dry samples were then crushed in an agate mortar to pass through a 0.125-mm screen, and 5 g of each sample was used in subsequent analyses. The samples were compressed at 37.8 Mpa for 2 min. to produce 32-mm-thick disks, and then the element concentrations were measured by means of X-ray fluorescence spectroscopy using an Axios Advanced PW4400 spectroscope.

The soil’s particle-size composition was determined using myeloperoxidase [21]. Soil organic matter content was determined after passing the samples through a #100 mesh to remove coarse particles, followed by heating at 105±2°C for 6 h to determine the loss of mass. The gas method was used to measure the CaCO_3_ content [21]. The Fe^3+^ and Fe^2+^ contents were determined using the colorimetric method and SOM was determined using the potassium dichromate (K_2_Cr_2_O_7_) oxidation method [21].

The mineral composition of the soil samples was determined using several common indices. The silicate coefficient (*K*_*i*_) equals the ratio of SiO_2_ to Al_2_O_3_. The redox condition was represented by the ratio of FeO to Fe_2_O_3_. The degree of differentiation among alkaline earth metals was represented by the ratio of CaO to MgO. The relationship between mineral composition and granularity was represented by the ratio of K_2_O to Na_2_O. We also calculated the chemical alteration index (*CIA*) to represent the degree of chemical weathering, as follows,

$$CIA\;=\;\left\{x\left(Al_{2}O_{3}\right)\;/\;\left[x\left(Al_{2}O_{3}\right)\;+\;x\left(CaO*\right)\;+\;x\left(Na_{2}O\right)\;+\;x\left(K_{2}O\right)\right]\right\}\times 100\%$$

where *x* represents the molar fraction of each oxide, and CaO* is the CaO content of silicate cement (which varied among the samples).

## Results

### 3.1. Weathering process of carbonate nodules and paleosols

**Fig 2:**
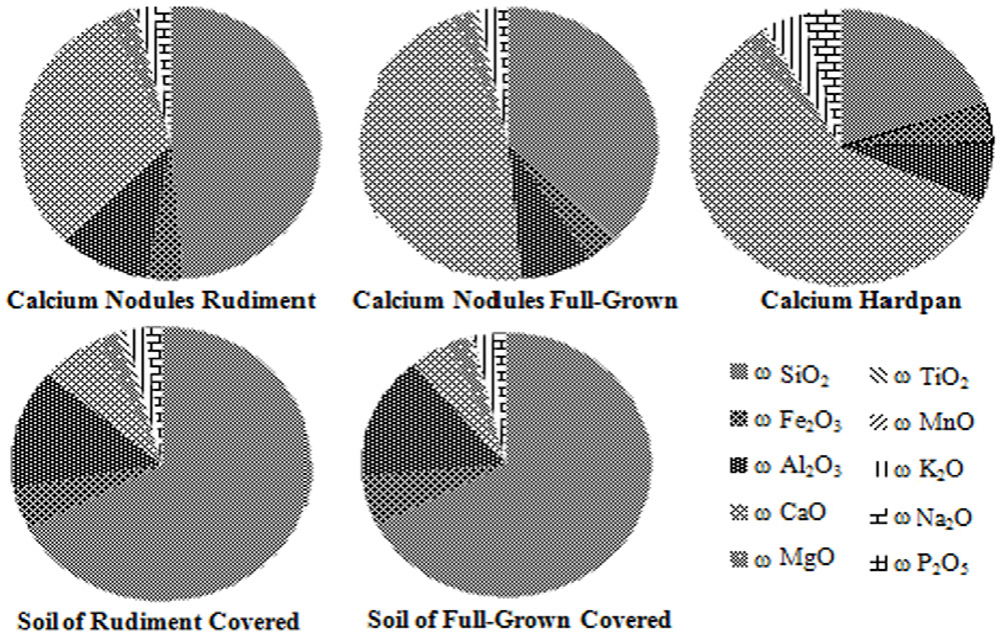
Chemical compositions of the calcium nodules and of the paleosol in the five soil type–vegetation cover combinations.

The SiO_2_ content was higher in the paleosol than in the calcium nodules, which had a high CaO content. The CaO content was higher in the immature soil with a sparse vegetation cover than in the more mature soil both in Luochuan and Lantian. and the development of calcium nodules along the evolutionary sequence shows gradual enrichment of CaO from an immature soil with a sparse vegetation cover to a soil with a dense vegetation cover, and then to a calcium hardpan (Fig 2). We further calculated an index of composition variation (*ICV*),

$$ICV\;=\;\left(Fe_{2}O_{3}+\;K_{2}O\;+\;Na_{2}O\;+\;CaO\;+\;MgO\;+\;MnO\;+\;TiO_{2}\right)\;/\;Al_{2}O_{3}$$

**Fig 3:**
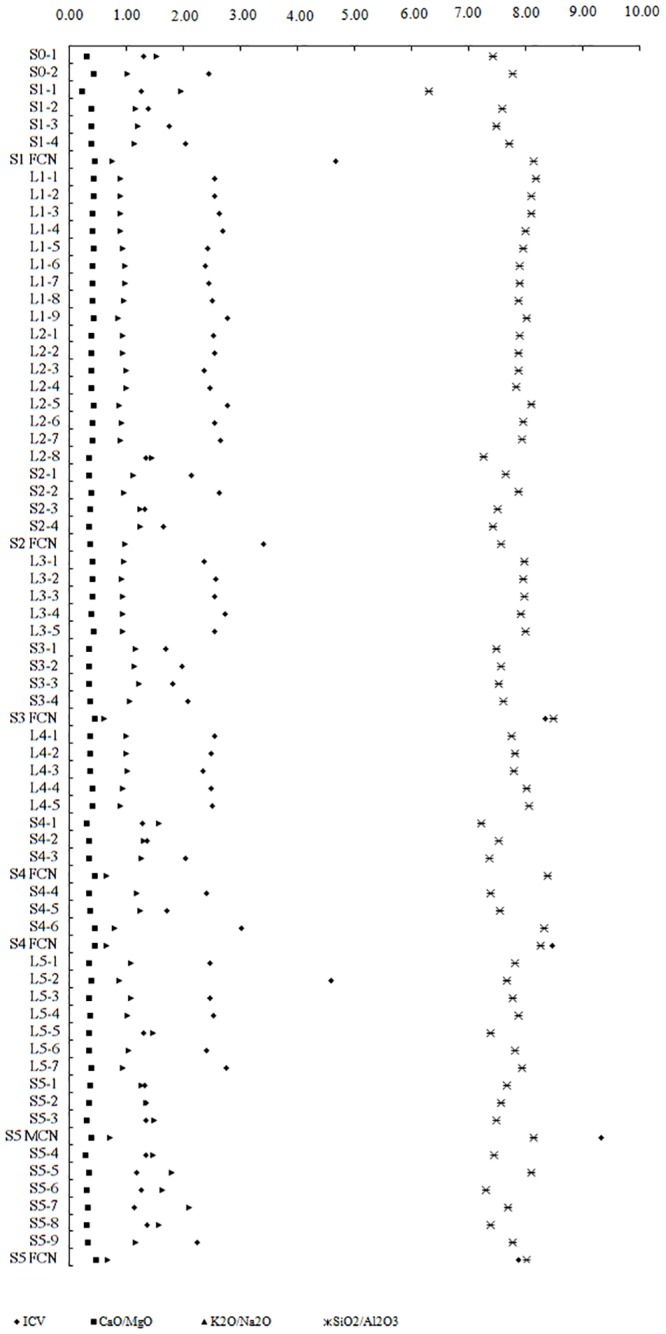
Composition ratios for the soil and carbonate nodule samples. ICV, index of composition variation.

It shows that *K*_*i*_ characterizes the relationship between quartz (SiO_2_) and feldspar (Al_2_O_3_), with high values of SiO_2_ /Al_2_O_3_ indicating a cold, dry climate (such as that at Luochuan) based on the degrees of weathering and leaching (Fig 3). The CaO/MgO ratio reflects the process of migration of these oxides during weathering, and the K_2_O/Na_2_O ratio represents the overall degree of similarity of the mineral composition in the loess, which relates mainly to the size and grain mineral composition of the grains. This reflects a higher degree of controlled by the internal cohesiveness of the loess particles of the loess in the Luochuan section than in the Lantian section.

**Fig 4:**
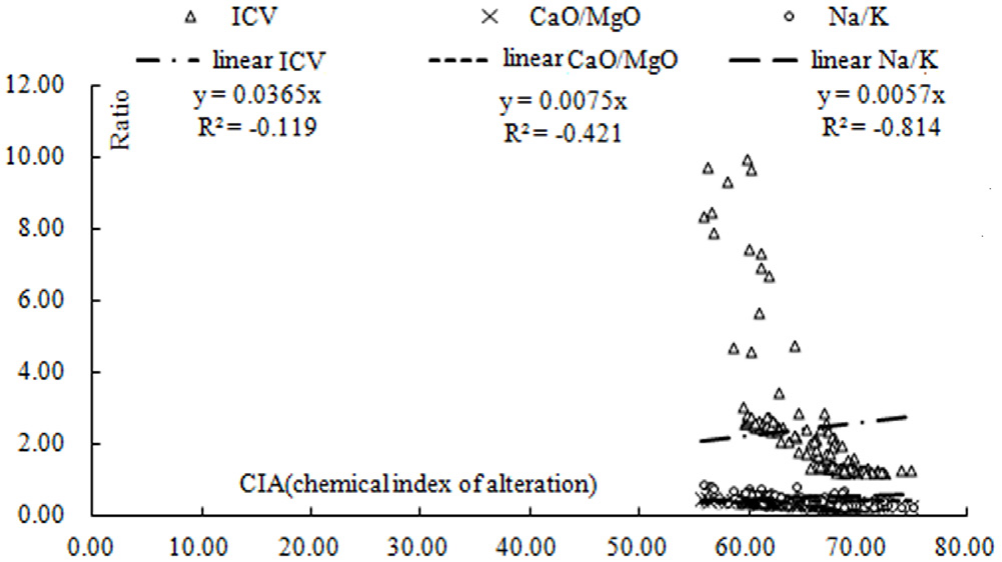
Weathering characteristics and eluvial nature of the paleosol. CIA, chemical index of alteration; ICV, index of composition variation.

The loess characteristics suggest a dry and cold climate in which both rainfall and temperatures were low. Weathering was, therefore, primarily through physical means, and weak chemical weathering resulted in dissolution of the native, clastic carbonates (Fig 4). The loess also contains biogenic speciments (e.g., anaerobic bacteria, fungi, and nematodes), and rod-shaped calcites that likely formed under dry conditions. We dissolved the samples in 1 mol L^-1^ acetic acid, and the amount of acid-insoluble CaO (the difference between total CaO content and the CaO dissolved in the acetic acid) represents the residual calcium silicate, which was significantly higher in the loess than in the paleosol, as was the case for the carbonate content. The loess can thus be considered to be eluvial in origin.

**Fig 5:**
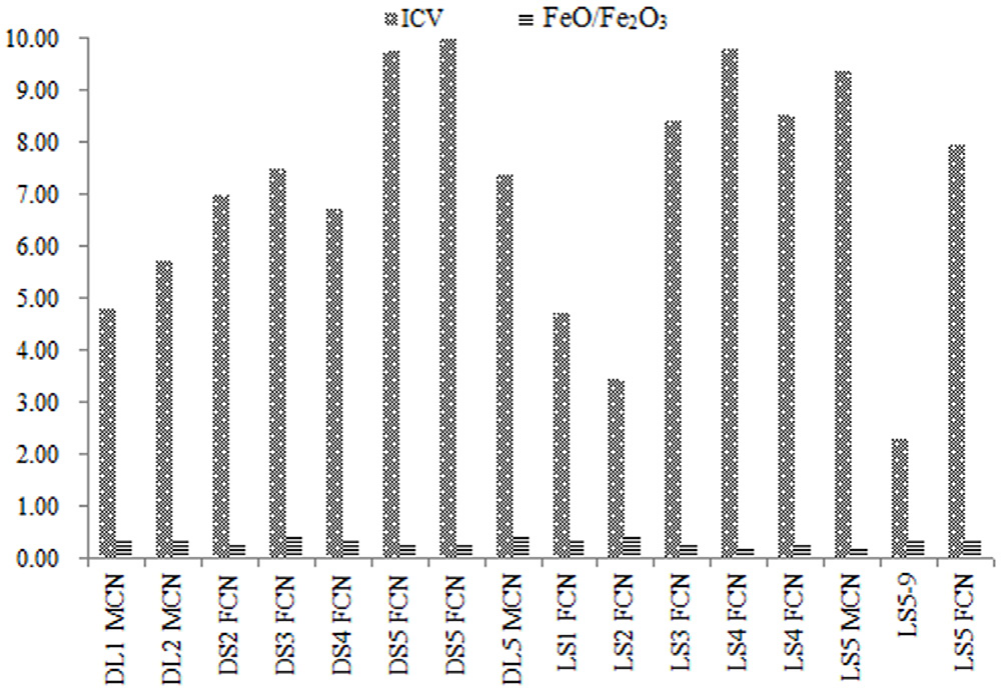
Histogram illustrating the recycling of calcium nodules (ICV, index of composition variation) and redox conditions (FeO/Fe_2_O_3_) in the loess.

**Fig 6:**
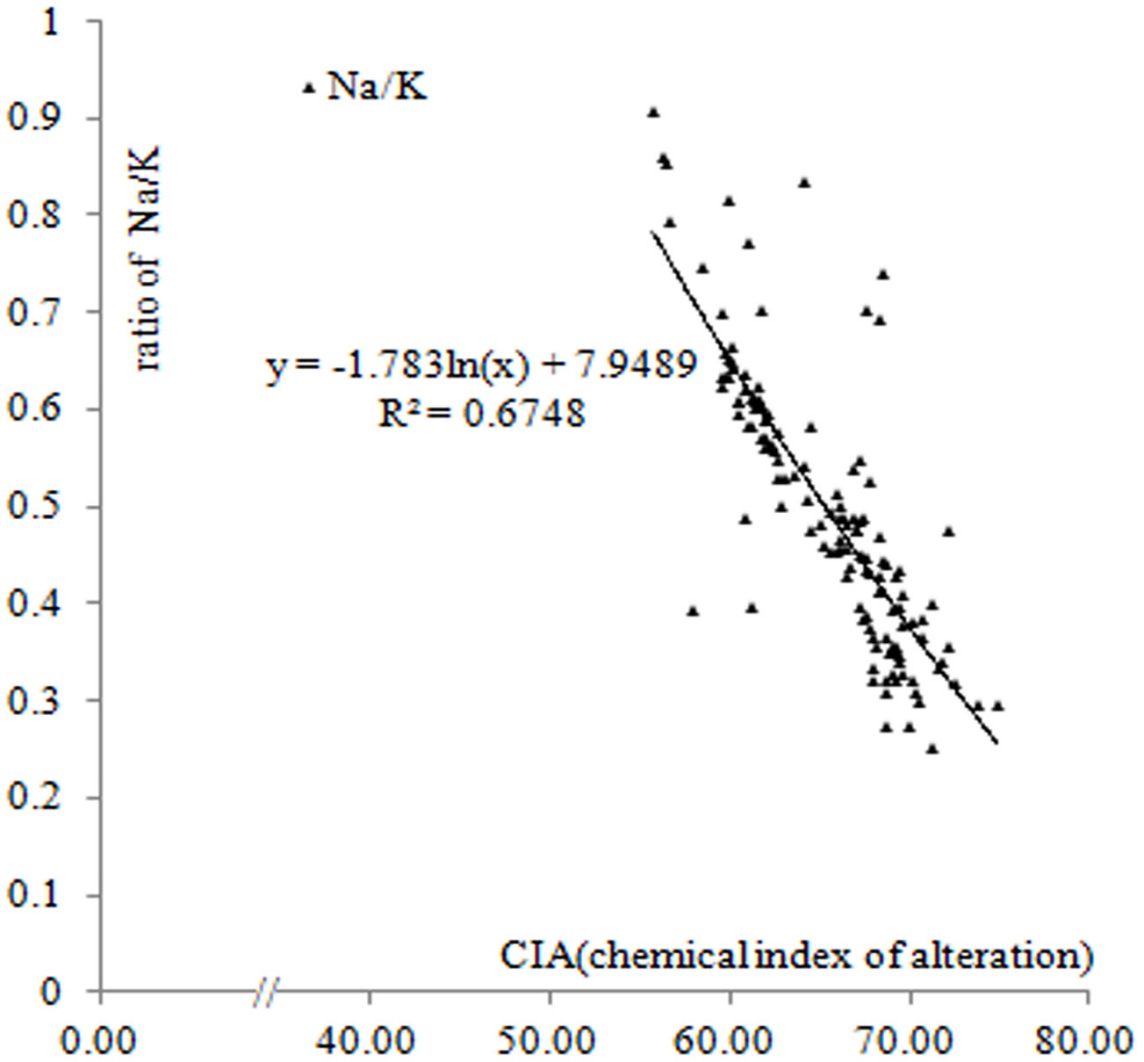
Relationship between the chemical index of alteration (CIA) of the paleosol and carbonate nodules from the Luochuan section and the Na/K ratio.

*ICV* represents the effect of calcium nodule recycling on transformation of the sediment composition; an ICV> 5 reflects a small effect of calcium deposition and recirculation. The FeO/Fe_2_O_3_ ratio, which reflects the redox conditions during loess formation, is closely related to both climate and the environment. Fig 5 shows that the Lantian samples, which were obtained at the edge of the Weihe River, had a higher degree of oxidation, suggesting more humid conditions than those those existed during formation of the Luochuan section. These values reflect the chemical weathering characteristics, and based on the CIA values in our loess samples (55 to 70), the samples appear to be of glacial origin (Fig 6).

Weathering intensity was relatively strong in the paleosol, and it was a chemical weathering that involved calcium leaching and the formation of residual silica. Using the weakly weathered loess as a benchmark, chemical weathering of the paleosol involved not only primary carbonate leaching, but also decomposition of calcium silicate. When temperatures and humidity were intermediate between glacial periods and periods with high temperature and high rainfall, the native carbonates of the paleosol dissolved completely, and this was accompanied by strong biological activity and silicate weathering (especially for calcium feldspar). Consequently, the carbonate content of the paleosol was much lower than that of the loess soil, and the content of acid-insoluble CaO was highest in the loess. A large number of soluble ions and salts were produced in the paleosol after migration that resulted from silicate weathering, leaving behind insoluble Si, Al, and Fe. The weathering stages of the paleosol and loess were thus different, with calcium being strongly leached in the paleosol, accompanied by the formation of residual silicon and aluminum phases.

### 3.2. Carbonate nodules as a proxy for environmental change

**Fig 7:**
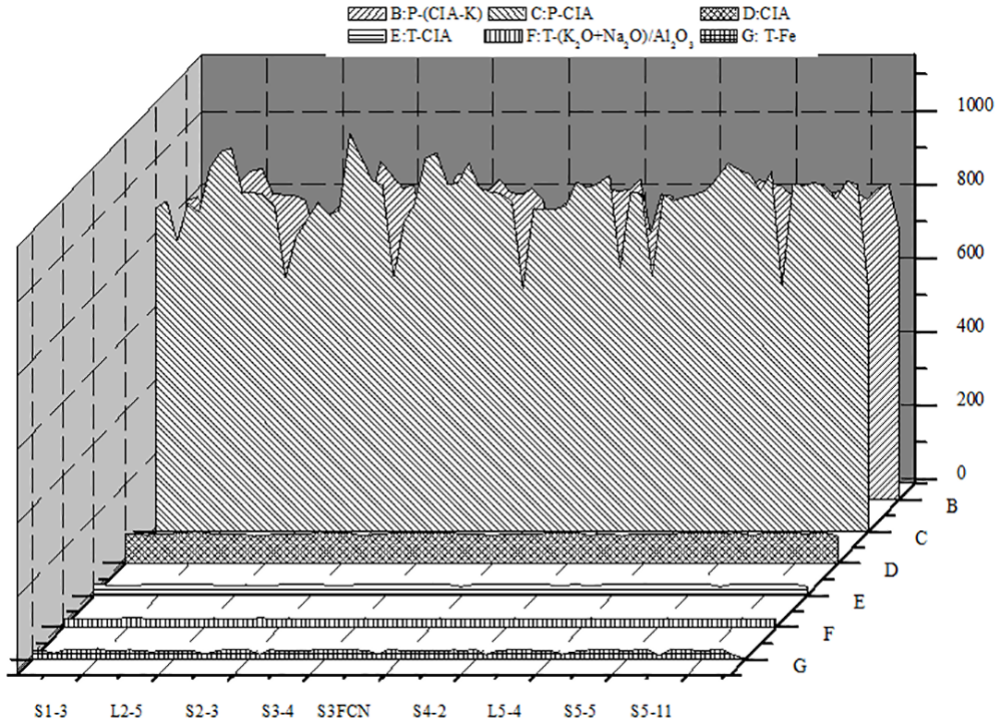
The estimated temperature and precipitation based on the different weathering indices forthe Luochuan section section. CIA, chemical index of alteration; CIA-K, CIA without K.

**Fig 8:**
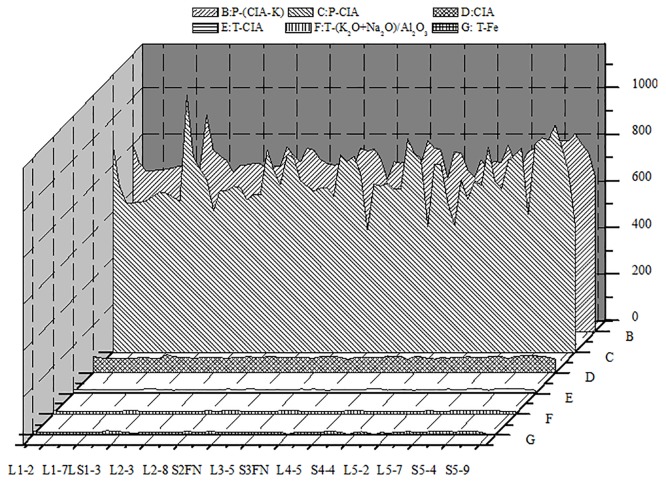
The estimated temperature and precipitation based on the different weathering indices for the Lantian section. CIA, chemical index of alteration; CIA-K, CIA without K.

**Fig 9:**
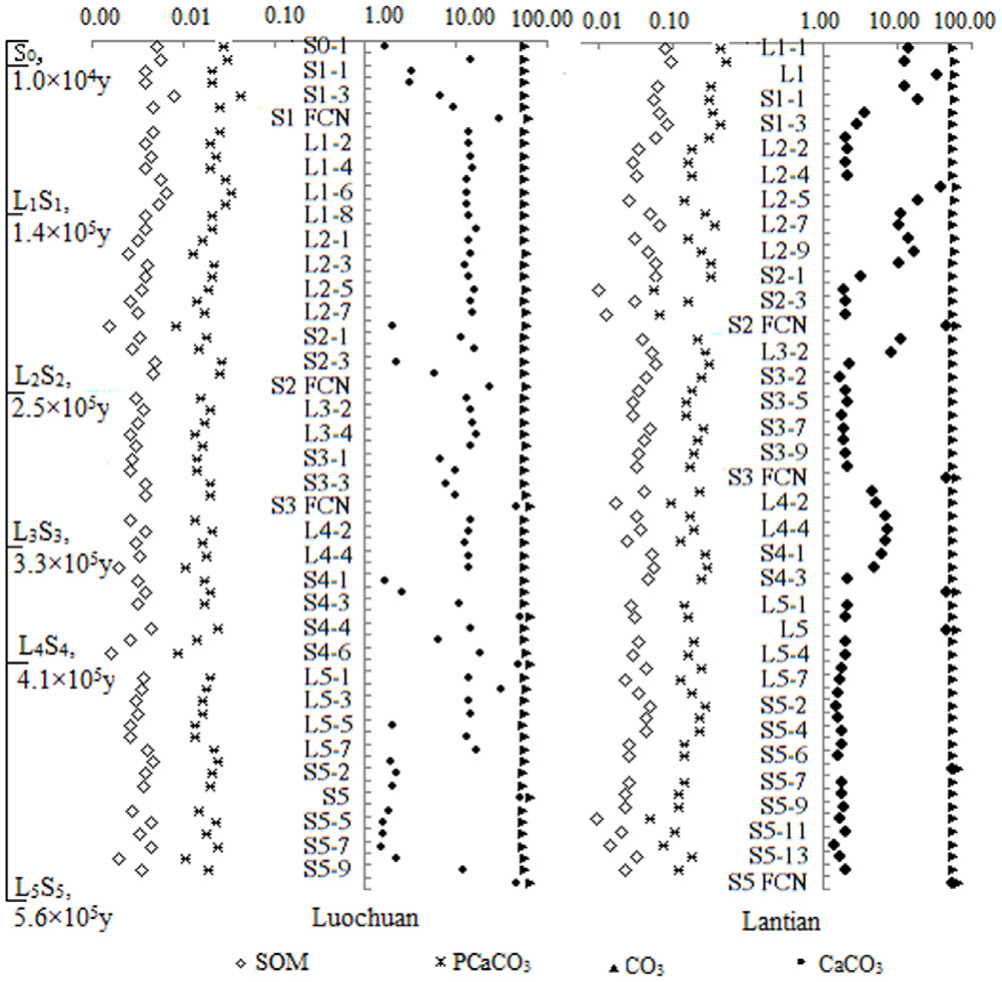
Changes in paleosol organic matter and the content of three forms of carbonate (PCaCO_3_, which represents CO_3_, and CaCO_3_). SOM, soil organic matter.

We further investigated the relationships among temperature, precipitation, and the weathering indices in reference to the precipitation equations [7–11]. Total annual precipitation ranged from 150 to 200 mm at Lantian and from 100 to 250 mm at Luochuan. Precipitation estimated from the fundus carbonate nodules ranged from 80 to 200 mm, which is 20 to 170 mm less than precipitation in the same paleosol at Lantian and Luochuan. The calcium nodules indicated that the temperatures during formation of the soil strata did not vary by more than 2 to 3°C (Figs 7 and 8) Organic matter in the paleosol was present at a concentration of 0.01% to 0.10%, but decreased over time (Fig 9). The relatively high total organic carbon content of the paleosol represents the development of a climate suitable for significant plant growth, but such an increase also decreases the rate of soil development, which is not conducive to biological growth over the long term. In addition, the amount of weathering residues and soil CaCO_3_ decreased over time, suggesting the existence of a climate–soil feedback mechanism.

## Discussion and Conclusions

Based on the characteristics of the Lantian and Luochuan calcium nodules, it is evident that the carbonate nodules changed in response to changes in the paleoclimate and soil mineral composition.

The potential for migration of ions, and enrichment thereof, depends strongly on the chemical properties of the sediments, as well as on geological processes such as weathering. In comparison with the Lantian profile, the Luochuan loess can be considered as a more weakly alkaline and oxidizing medium based on the measured pH and Eh. In the Luochuan section, pH increased from the base to the top, while Eh decreased, which is consistent with evidence that the Pleistocene climate became increasingly arid [20]. The loess profile (including soil and buried regolith) had pH values ranging from 7.32 to 8.52, with an average of 8.37. Only two samples were collected from the upper paleosol and they had pH values less than 8.00 and Eh values greater than 500 mV. The pH values of the remaining samples were between 8.20 and 8.50 and Eh was between 430 and 474 mV.

The CaCO_3_ content of the loess would have changed as a result of changes in pH and Eh. When the climate was relatively humid, a significant proportion of the CaCO_3_ was reduced by a flow of alkaline water, leaching, and a concomitant decrease in pH and increase in Eh. This suggests that changes in the distribution of pH and Eh in the loess profile are closely related to climate changes. This can be explained if Ca and Mg in the surface runoff after precipitation resulted in carbonate deposition, and CaCO_3_ and MgCO_3_, respectively, entered the paleosol in rainwater. During paleosol development, the climate was warm and humid, and it was during this time that carbonate was leached; therefore, we have only considered the effect of precipitation during the time of loess accumulation. The average annual rainfall during the time of loess accumulation was approximately 20 to 44 mL/cm^2^ per year in the Luochuan section [15]. The remainder became runoff and infiltrated the soil, thereby facilitating carbonate precipitation within the loess at an annual rate of 0.06 to 0.11 mL/cm^2^. The rate of loess accumulation in the Luochuan section was 9.4 mL/cm^2^, versus 59.8 mL/cm^2^ in the Lantian section. Therefore, rainfall during the accumulation of the loess caused the precipitation of 0.58 to 1.17 mg of carbonate annually.

Thus, a lower pH and higher Eh would reflect a period with relatively high humidity and warmth, whereas a high pH and lower Eh would reflect a period with relatively dry and cold conditions. The pH and Eh of the paleosol and loess layers suggest that the climatic conditions during the time of paleosol development were wetter than those during the period of loess accumulation.

The erosion rate during a geological period can be estimated from such rates determined from one or more strata during that period. The Lantian and Luochuan profiles show two to three layers of dark loess soil that likely correspond to known 10-ka denudation events in the Holocene Epoch [20,22]. Based on the stratigraphy of the Luohe Terrace, we concluded that there were five major developmental stages since the Middle Pleistocene in the Luochuan part of the Loess Plateau. According to Strahler’s drainage theory [23], the Luochuan part of the Loess Plateau has entered a primary period of erosion, with an average annual erosion modulus of 272.6 t/km^2^ since stratum S5 developed (~550 ka BP). Based on the universal soil-loss equation [24], the current erosion modulus is 4389 t/km^2^. The average rate of erosion has also increased; the average erosion intensity since stratum S2 developed is 1.8 times greater than the earlier rate from 550 ka to 250 ka BP. Importantly, these results also show that the environment and the climate changed significantly during these periods.

The carbonate content of the loess–paleosol sequences of the Luochuan Plateau in the Loess Plateau was controlled by an alternating wet and dry paleoclimate during the Quaternary. High rainfall and humid conditions increased surface runoff and the erosion of native, clastic carbonates and increased water infiltration and leaching of the paleosol. The sediment characteristics (e.g., grain size, sediment components) directly affect the leaching of surface elements and their migration through the soil. However, decreased rainfall and dry conditions led to carbonate supersaturation and the precipitation of Ca(HCO_3_)_2_ from the soil solution, leading to the formation of secondary carbonates in the loess. Given that the earliest loess soil had calcium nodules at its base, and that their formation was closely related to the contemporaneous climate, we conclude that this depth provides an indication of the timing of rainfall and the depth distribution of leaching conditions. The paleoclimate, geomorphology, and soil mineral constituents influenced the surface vegetation and its evolution, and thereby directly influenced changes in the soil. The evolution of surface vegetation slowed during the periods when carbonate nodules formed, and both the number of plant species and their abundance (measured by the quantity of pollen and spores) decreased. The carbonate content therefore reflects the degree to which the soil was eluviated and, to a certain extent, indicates the changes in soil moisture [25].

Through this investigation, we conclude that calcium nodules can be regarded as an efficient proxy of Quaternary paleoclimate change in China’s Loess Plateau. The changes in carbonate content in the loess and paleosol sequences were controlled by alternating dry and wet climatic conditions. Nodule formation conditions were directly affected by the leaching and migration of elements. The oldest loess sequence developed calcium nodules at its base, and their formation was closely related to the rainfall and leaching characteristics of the paleoclimate. The paleoclimate, soil minerals and vegetation types directly influenced changes in the soil.

## References

[1] Lee H, French C, Macphail R (2014) Microscopic examination of ancient and modern irrigated paddy soils in South Korea, with special reference to the formation of silty clay concentration features. Geoarchaeology 29(4): 326–348.

[2] Wanas H, Abu El-Hassan M (2006) Paleosols of the upper cretaceous–lower tertiary maghra El-Bahari formation in the northeastern portion of the Eastern Desert, Egypt: their recognition and geological significance. Sedimentary Geology 183: 243–259.

[3] Magaritz M, Kaufman A, Yaalon D (1981) Calcium carbonate nodules in soils: ^18^O/^16^O and ^13^C/^12^C ratios and ^14^C contents. Geoderma 25: 157–172.

[4] Retallack GJ, James WC, Mack GH, Monger HC (1993) Classification of paleosols: Discussion and reply. Geological Society of America Bulletin 105: 1635–1637.

[5] Mack GH, James WC, Monger HC (1993) Classification of paleosols. Geological Society of America Bulletin 105: 129–136.

[6] Nettleton W, Olson C, Wysocki D (2000) Paleosol classification: problems and solutions. Catena 41: 61–92.

[7] Lu Y, Sun J, Zhang J, Li P (2006) Estimation of paleo-temperature with total iron content in loess. Arid Land Geography 28: 450–454. (in Chinese with English summary)

[8] Sheldon ND, Retallack GJ, Tanaka S (2002) Geochemical climofunctions from North American soils and application to paleosols across the Eocene–Oligocene boundary in Oregon. Journal of Geology 110: 687–696.

[9] Retallack GJ, Huang C (2010) Depth to gypsic horizon as a proxy for paleoprecipitation in paleosols of sedimentary environments. Geology 38: 403–406.

[10] Kraus MJ, Riggins S (2007) Transient drying during the Paleocene–Eocene Thermal Maximum (PETM): analysis of paleosols in the Bighorn Basin, Wyoming. Palaeogeography, Palaeoclimatology, Palaeoecology 245: 444–461.

[11] Ouyang C, Li B, Ou X, Wen X, Zeng L, Yang Y, et al (2007) Chemical Weathering of the Milanggouwan Paleosols in the Salawusu River valley and their paleoelimatie implications during the Last Interglaeial Period. Acta Geographica Sinica 62: 518–528. (in Chinese with English summary)

[12] Maynard J (1992). Chemistry of modern soils as a guide to interpreting Precambrian paleosols. Journal of Geology: 100(3): 279–289.

[13] Wen Q (1989) Geochemistry in Chinese loess. Beijing: China Science Press.

[14] Guo Z., Ruddiman WF, Hao Q, Wu H, Qiao Y, Zhu R, et al (2002) Onset of Asian desertification by 22 Myr ago inferred from loess deposits in China. Nature 416: 159–163. 11894089 10.1038/416159a

[15] Liu L, Yin Q, Wu H, Guo Z (2010) Carbon isotopic compositions of pore and matrix carbonates in carbonate nodules, and origin of carbonate formation. Chinese Science Bulletin 55: 2926–2929.

[16] Emiliani C (1955) Pleistocene temperatures. Journal of Geology 63: 538–578.

[17] Shackleton NJ, Opdyke ND (1973) Oxygen isotope and palaeomagnetic stratigraphy of Equatorial Pacific core V28-238: oxygen isotope temperatures and ice volumes on a 10^5^ year and 10^6^ year scale. Quaternary Research 3: 39–55.

[18] Han J, Keppens E, Liu T, Paepe R, Jiang W (1997) Stable isotope composition of the carbonate concretion in loess and climate change. Quaternary International 37: 37–43.

[19] Gallet S, Jahn B-M, Torii M (1996) Geochemical characterization of the Luochuan loess-paleosol sequence, China, and paleoclimatic implications. Chemical Geology 133: 67–88.

[20] Liu T (2004) Loess and Environment. Beijing; Science Press.

[21] Liu L, Zhou X, Yu Y, Guo Z 2011 The natural vegetations on the Chinese loess plateau: the evidence of soil organic carbon isotope. Quaternary Sciences 31: 506–513.

[22] Ballestrero T, Herzog B, Thompson G (2006) Monitoring and sampling the vadose zone In: Nielsen David M. (editor), Practical handbook of ground-water monitoring. Lewis Publishers, Inc. Chelsea, Michigan, 207–247.

[23] Strahler AN (1957) Quantitative analysis of watershed geomorphology, Transactions of the American Geophysical Union 38 (6): 913–920.

[24] Shuang GS, Gan ZM (2005) Preliminary research on valleys and gullies development process and quantity of soil erosion (Changes in Luochauan Loess Plateau region since Late Middle-Pleistocene. Journal of Soil and Water Conservation 19: 109–113.

[25] Lu H, An Z (1999) Comparison of grain-size distribution of red clay and loess–paleosol deposits in Chinese loess plateau. Acta Sedimentologica Sinica 17: 226–232.

